# White Matter, Age, APOE-e4, and Episodic Memory in Midlife Adults at Heightened Risk for Alzheimer’s Disease

**DOI:** 10.1093/geroni/igaf122.4272

**Published:** 2025-12-31

**Authors:** Chadsley Wessinger, Jennifer Etnier, Kyoung Shin Park, Samantha DuBois, Samuel Kibildis, Jarod Vance, Brittany Armstrong, Alexis Slutsky-Ganesh

**Affiliations:** University of North Carolina at Greensboro, Greensboro, North Carolina, United States; University of North Carolina at Greensboro, Greensboro, North Carolina, United States; Emory University School of Medicine, Atlanta, Georgia, United States; Appalachian State University, Boone, North Carolina, United States; Athens-Clarke County Unified Government, Athens, Georgia, United States; Longwood University, Farmville, Virginia, United States; University of North Carolina at Greensboro, Greensboro, North Carolina, United States; North Carolina Agricultural and Technical State University, Greensboro, North Carolina, United States

## Abstract

Episodic memory (EM) decline is an early cognitive symptom of Alzheimer’s disease (AD). White matter microstructural integrity (WMI) supports EM, but it is unclear if AD risk factors of age and apolipoprotein epsilon-4 genotype (APOE-e4) influence the relationship between WMI and EM at midlife prior to AD symptom onset. We investigated if age and APOE-e4 carriage independently moderated the relationship between WMI and EM in cognitively normal middle-aged adults with a family history of AD (FH+). Participants (N = 109[84% female]; Mage=56.2±6.0 years) provided saliva samples for APOE-e4 genotyping (e4 non-carriers/carriers=61/48) and completed diffusion tensor imaging (DTI) and cognitive testing. DTI scalars—mean diffusivity (MD), axial diffusivity (AxD), and radial diffusivity (RD)—were estimated in the fornix and hippocampal cingulum (CGH), tracts critical for EM. EM was assessed with Auditory Verbal Learning Task Immediate Recall (AVLT-IR) and Delayed Recall (AVLT-DR). Moderated regression models were fitted; significant (p<.05) and near-significant (p<.10) interactions were followed by simple slope analyses. No WMI*APOE-e4 carriage interactions were significant (p’s>.10). Age moderated the WMI–AVLT-IR associations for CGH AxD (left: p=.027, right: p=.011), MD (left: p=.011, right: p=.005), and RD (right: p=.044); and the WMI–AVLT-DR associations for CGH AxD (left: p=.009, right: p=.010) and MD (left: p=.014, right: p=.011). In the oldest of participants (Mage=62.7±1.9 years), bilateral CGH AxD and MD negatively predicted AVLT-IR (p’s=.011-.023) and AVLT-DR (p’s=.012-.062) performance. In cognitively normal middle-aged adults with FH+, negative relationships between CGH WMI and EM were observed, suggesting the possibility of pre-clinical associations between brain health and cognitive behavior.

